# Alternative Destination Transport? The Role of Paramedics in Optimal Use of the Emergency Department

**DOI:** 10.5811/westjem.2016.9.31384

**Published:** 2016-10-04

**Authors:** Michael M. Neeki, Fanglong Dong, Leigh Avera, Tan Than, Rodney Borger, Joe Powell, Reza Vaezazizi, Richard Pitts

**Affiliations:** * Arrowhead Regional Medical Center, Department of Emergency Medicine, Colton, California; † Western University of Health Sciences, Graduate College of Biomedical Sciences, Pomona, California; ‡ City of Rialto Fire Department, Rialto, California

## Abstract

**Introduction:**

Alternative destination transportation by emergency medical services (EMS) is a subject of hot debate between those favoring all patients being evaluated by an emergency physician (EP) and those recognizing the need to reduce emergency department (ED) crowding. This study aimed to determine whether paramedics could accurately assess a patient’s acuity level to determine the need to transport to an ED.

**Methods:**

We performed a prospective double-blinded analysis of responses recorded by paramedics and EPs of arriving patients’ acuity level in a large Level II trauma center between April 2015 and November 2015. Under-triage was defined as lower acuity assessed by paramedics but higher acuity by EPs. Over-triage was defined as higher acuity assessed by paramedics but lower acuity by EPs. The degree of agreement between the paramedics and EPs’ evaluations of patient’s acuity level was compared using Chi-square test.

**Results:**

We included a total of 503 patients in the final analysis. For paramedics, 2 51 (49.9%) patients were assessed to be emergent, 178 (35.4%) assessed as urgent, and 74 (14.7%) assessed as non-emergent/non-urgent. In comparison, the EPs assessed 296 (58.9%) patients as emergent, 148 (29.4%) assessed as urgent, and 59 (11.7%) assessed as non-emergent/non-urgent. Paramedics agreed with EPs regarding the acuity level assessment on 71.8% of the cases. The overall under- and over-triage were 19.3% and 8.9%, respectively. A moderate Kappa=0.5174 indicated moderate inter-rater agreement between paramedics’ and EPs’ assessment on the same cohort of patients.

**Conclusion:**

There is a significant difference in paramedic and physician assessment of patients into emergent, urgent, or non-emergent/non-urgent categories. The field triage of a patient to an alternative destination by paramedics under their current scope of practice and training cannot be supported.

## INTRODUCTION

Expanding the role of emergency medical services (EMS) has become an emerging topic of conversation given the need to expand local access to healthcare resources for communities and their residents. It is estimated that in 2011, national emergency department (ED) visits totaled 131 million, or 421 ED visits per 1,000 population.^1^ The total number of these ED visits that could be considered non-urgent has been difficult to determine, with numbers ranging from 4.8% to 90% of visits.^2^ The criteria used to determine non-urgency of a patient presentation have proven difficult to establish with multiple reports using different definitions.

California Health and Safety Code Division 2.5, section 1797.52, requires that all patients who call 911 be taken to an acute hospital with a basic or comprehensive ED to receive further evaluation by medical staff.^3^ However, it has been proposed that some 911 calls for low-acuity conditions could potentially be diverted to non-ED settings such as urgent care clinics or primary care offices, possibly reducing the crowding and long wait time seen in many EDs and, as a result, reduce the cost of healthcare.^4^

In July 2013, a report published by the Institute for Population Health Improvement, University of California Davis Health Systems underlined possible changes to the current California EMS system. Included in this report was the proposal that patients with specified conditions not needing emergency care could be transported to non-ED locations or alternative destination transport. The alternative destination locations listed included mental health facilities, urgent care clinics or primary care offices. 4 Multiple published national reports estimate that 11% to 61% of ambulance transports may not require immediate care in the ED.^5^ Based on this report, the Emergency Medical Services Authority (EMSA) has initiated pilot programs in California to study the feasibility of alternative transportation. As of 2016, four pilot programs have been approved to study alternative transportation destinations in California.^6^,^7^

In those circumstances where EMS providers encounter patients who do not need advanced life support (ALS) level of care or evaluation at an ED, transportation to an alternative destination may be more cost effective. EMS systems with proper resources along with close medical oversight may be good candidates for implementation of such a program. However, the majority of research in this area has concluded that there is currently insufficient evidence to support widespread implementation of non-transport and alternative destination protocols.^5^,^8^,^9^

This pilot study aims to assess the accuracy of the paramedic’s assessment of a patient’s acuity level and identify areas of improvement in prehospital patient care. In addition, the findings from this pilot study could be used to address any deficiencies in paramedic training, which in turn could strengthen the programs for alternative transport destinations.

## METHODS

### Study Design and Setting and Selection of Participants

This is a prospective double-blinded study analyzing the responses recorded by paramedics versus licensed emergency physicians (EP) of patients transported to Arrowhead Regional Medical Center (ARMC) by licensed paramedics with Rialto Fire Department (RFD) between April 2015 and November 2015. RFD’s California state-licensed paramedics serve a population of 101,109 in a 22.37 square mile urban setting located in San Bernardino County, the largest county in the United States. RFD responded to 7,617 calls for medical assistance in 2015. The RFD has 45 paramedics trained to provide ALS, including administering medications, establishing vascular access, advanced airway placement, cardiac rhythm interpretation and defibrillation. During the study period, RFD ambulances transported 1,720 patients to ARMC, of which 505 were randomly selected for this study.

ARMC is a 456-bed acute care hospital in Colton, California. ARMC is the only American College of Surgeons-verified Level II trauma center serving San Bernardino County.^10^ ARMC ED is the second busiest in California and has an annual volume of more than 116,000 visits.^10^ Additionally, more than 12 ground and air providers transport patients to ARMC. These providers operate within the 20,000 square miles of San Bernardino County and provide coverage for a mix of urban and rural communities with a total population of over 2.1 million.^11^,^12^

The EPs responsible for collecting data were board-certified in emergency medicine or senior level emergency medicine residents with completion of three or more years of training. The institutional review board of ARMC approved this study.

### Data Collection and Processing

We calculated the degree of agreement between the paramedics’ and EPs’ evaluation of emergent, urgent, and non-emergent/urgent patient presentations transported by paramedics. Emergent conditions were defined as requiring immediate attention with threat of life. Urgent conditions were defined as requiring immediate attention without threat of life that could go to a non-ED facility. Lastly, non-emergent/non-urgent was defined as patients not requiring transportation.

The primary outcome was agreement on the acuity level assessed by paramedics and EPs, respectively. Agreement was defined as the same acuity level being assessed by paramedics and EPs. Under-triage was defined as a lower acuity assessed by paramedics but a higher acuity by EPs. Over-triage was defined as a higher acuity assessed by paramedics but a lower acuity by EPs. To decrease the variability of the outcome, this study was limited to one group of paramedics with similar education, regulatory oversight, and medical supervision. Furthermore, the geographic region and population sampling was also limited to one particular area.

**Figure 1:**
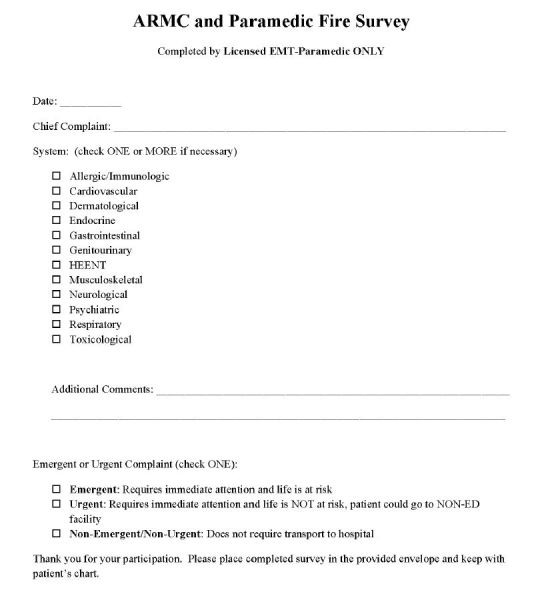
Evaluation form used by paramedics to assess patient acuity.

**Figure 2:**
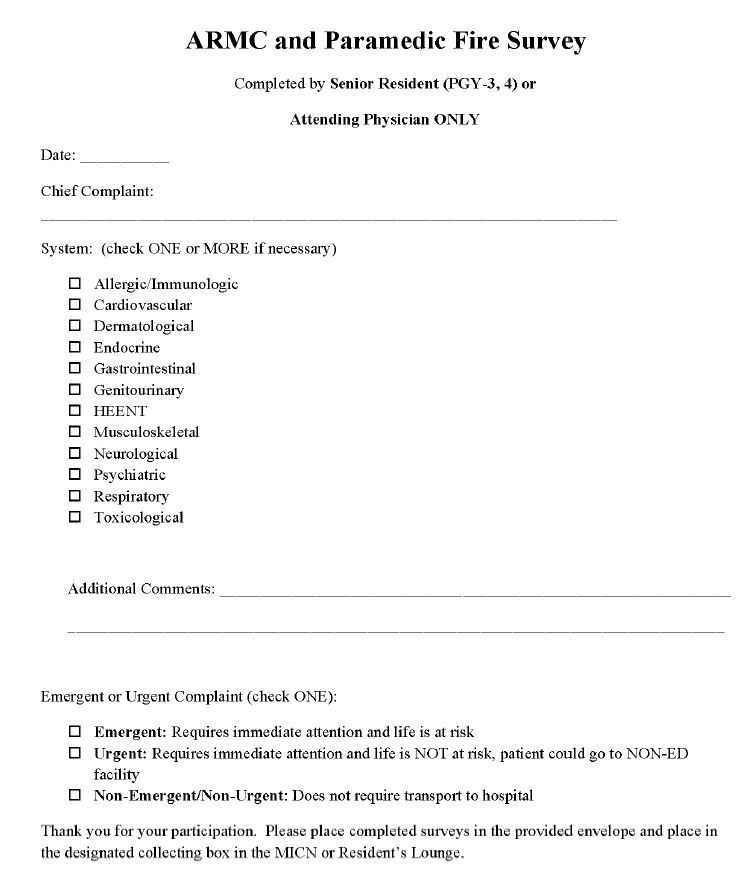
The Evaluation Form-Emergency Medicine provider

Upon evaluation of each patient in the field, RFD paramedics completed an evaluation form (Figure 1) indicating the chief complaint of the patient being transported, the body system affected, and the decision as to whether there was an emergent/urgent versus non-emergent/non-urgent condition. Each form was then placed in a sealed envelope and handed to the receiving EPs along with a corresponding blank evaluation form (Figure 2). The receiving EP would then complete the form immediately after physical evaluation and place both surveys in a large sealed envelope. The receiving EP had no knowledge of the responses recorded by RFD paramedics.

### Statistical Analysis

We conducted all statistical analyses using the SAS software for Windows version 9.3 (Cary, NC). Descriptive statistics were presented as frequencies and proportions for categorical variable. We performed a crosstab analysis to assess the inter-rater reliability (Kappa statistic) between paramedics’ and EPs’ assessment on patients’ conditions. All statistical analyses were two-sided. We considered p-value <0.05 to be statistically significant.

## RESULTS

A total of 505 patients transported by EMS had surveys completed by both a paramedic and an EP who evaluated their acuity level and presenting chief complaint with the corresponding body system affected. Two surveys were excluded due to missing acuity evaluations by paramedics, which led to a final sample size of 503. Among these 503 patients, 251 (49.9%) were assessed to be emergent, 178 (35.4%) assessed as urgent, and the other 74 (14.7%) assessed as non-emergent/non-urgent by paramedics (Table 1). In comparison, the EPs assessed 296 (58.9%) patients as emergent, 148 (29.4%) as urgent, and 59 (11.7%) as non-emergent/non-urgent. Paramedics agreed with the EP regarding the acuity level assessment on 71.8% of the patient cohort. The overall under- and over-triage were 19.3% and 8.9%, respectively. There is a statistically significant difference between paramedics’ and EP’s assessment on patient’s acuity level (p<0.0001, Table 1).

We conducted a crosstab analysis to identify the inter-rater agreement between paramedics’ and the EPs’ assessment on the same cohort of patients (Table 1). The inter-rater Kappa statistics was 0.5174, which indicated moderate inter-rater agreement between paramedics’ and EPs’ assessment on the same cohort of patients (n=503).

We conducted three subgroup analyses to identify the discrepancy between paramedics’ and EPs’ evaluation on patients’ acuity level. The first subgroup analysis is considered as “over-triage,” in which paramedics evaluated patients at a higher acuity level but the EPs’ evaluations of the same cohort of patients were at a lower acuity level (Table 2). The four systems most frequently over-triaged by the paramedics were neurological (n=10, 22.2%), musculoskeletal (n=8, 17.8%), cardiovascular (n=6, 13.3%), and gastrointestinal (n=5, 11.1%).

The second subgroup analysis was considered as under-triage, in which paramedics evaluated patients as lower acuity level but EPs evaluated the same cohort of patients as a higher acuity level (Table 3). The four systems most frequently under-triaged by paramedics included musculoskeletal (n=25, 25.8%), gastrointestinal (n=20, 20.6%), neurological (n=14, 14.4%), and cardiovascular (n=13, n=13.4%).

The third and last subgroup analysis was considered as correct triage, where paramedics and EPs made the same assessment on the patient’s acuity (Table 4). The top four most frequently correct triaged systems assessed by paramedics were neurological (n=73, 20.2%), musculoskeletal (n=68, 18.8%), cardiovascular (n=59, n=16.3%), and gastrointestinal (n=54, 15%).

## DISCUSSION

The study aimed to determine the level of agreement between paramedics and EPs in their evaluation of the acuity of the patient and the physiological systems involved. Paramedics agreed with EPs on 71.8% of the patient cohort regarding the assessment of the acuity level. The overall over-triage rate was 8.9% and the under-triage rate was 19.3%. There is significant difference in paramedic and physician classification of the alternative destination for emergency evaluation. Based on this pilot study, there is room for improvement in evaluation of those urgent and non-emergent/non-urgent patients as assessed by paramedics.

Morganti et al explored the topic of expanding the range of EMS transport options and the difficulties posed by such a change in current policy.^5^ This included the question of whether EMS providers can accurately identify patients who can be safely managed in a non-ED setting. Of special concern was the under-triaging of patients seeking access to emergency medical care. The reported under-triage rate in the current study was 19.3%, which was consistent with previous findings by Morganti et al, where they reported a wide range of rates (3% to 32%) of EMS personnel failing to recognize the severity of patients’ problems.^5^ This current study contributes to the literature by listing the four most frequently under-triaged systems by paramedics.

It is our goal to use the data from this pilot study to attempt to institute further training for paramedics to distinguish potentially emergent conditions from the urgent or non-emergent/non-urgent to prevent under-triaging. For example, this may include decision rules depending on patient’s chief complaint, medical history, and age, which paramedics could use prior to labeling a patient as not requiring emergency room care.

However, many issues must be addressed to ensure the quality of alternative transportation and destination programs with patient safety as the upmost priority. EMS programs need to ensure implementation of continuous quality improvement of policies and procedures. One of the most essential steps is to develop educational programs for EMS personnel, physicians, and the community that encourage teamwork and improve compliance with established emergency medical dispatch criteria, particularly among the four systems most frequently associated with the 8.9% over-triage and 19.3% under-triage rate. Furthermore, any future studies and educational programs must ensure that alternative transportation and destination decisions are consistent with medical necessity and with consideration for patient preference and when the patient’s condition allows. This may call for more oversight and supervision of paramedics if alternative destination becomes a reality. EP supervision could be also implemented by using new technologies such as telemedicine.

A reduction in the use of EDs for non-emergency conditions, a practice that has often been suggested as contributing to the rising costs of healthcare, will ultimately require a multi-disciplinary approach. Diverse demographic and socioeconomic characteristics influence patients who contact 911 for ambulance transport, including a patient’s perception of his own acuity level and of how quickly an urgent care or primary care physician could address his complaint.^1^,^5^,^13^,^14^ Ultimately, the ED is a safety net for patients, especially for those without a primary care physician or patients with chronic medical problems who require treatments best addressed in the ED.^1^ Many proposed solutions have been discussed that could potentially avoid crowding and over-utilization of the ED. Part of the solution will require the involvement of case management, individualized care plans and information sharing.^8^,^14^,^15^

Telemedicine services may also offer opportunities for supporting patient management in prehospital care. With the introduction of smartphones over the past decade, telemedicine services have grown in the U.S. and many hospitals have implemented their use.. The ability to interact remotely with patients and EMS personnel is applicable in many ED settings. Because this method of communication provides instant, high-quality medical consultation, the result is an improvement in prehospital patient care. It is well recognized within the medical community, including professional emergency medicine organizations, that scientifically supported introduction of telemedicine services may improve quality of care. Adoption of this technology, however, has been slow and in some cases impeded by resistance from some state licensing boards and the reluctance of some private and government payers to reimburse for such services.^16^–^18^

Lastly, legislators will also have to support appropriate compensation for EMS systems based on patient evaluation and treatment as well as on alternative destination transport. Currently, the Centers for Medicare & Medicaid Services (CMS) only reimburses transport that is both “reasonable” and “medically necessary,” with the majority of Medicare-reimbursed ambulance calls involving transport to the ED.^5^ Additionally, payment for 911 service EMS ground transport is tied to level of service (BLS versus ALS), with private insurances following the lead on reimbursements made by CMS.^5^

## LIMITATIONS

This pilot study was subject to a few limitations that could potentially alter the outcome of our findings. We attempted to design a system that would allow EMS providers to make their evaluations without physician influence by having paramedics complete their forms prior to arrival to the ED. However, the current study does not take into account the influence on paramedics by the base station’s contact with a mobile intensive care nurse and/or EP. Even if prehospital influence from base contact were removed, there were instances when paramedics were unable to complete their forms prior to arrival due to patient acuity, shorter travel times, and need for patient treatments and interventions. The result was that paramedics may have filled out the forms after being directed by a nurse or physician to a specific area of the ED based on acuity. This initial evaluation by a nurse or physician would likely influence (bias) the paramedic’s evaluation of the patient.

Additionally, although EPs were directed to complete their evaluation forms after their own initial evaluation of the patient, many factors could alter their determination of acuity. The EP’s evaluation could have been influenced by the paramedic’s report and potential differential diagnoses offered, as well as by treatments administered (which may or may not have been necessary). The paramedic’s framing of his patient encounter could also have influenced the EP.

Other factors that could have caused a discrepancy between paramedics and EP evaluation include changing chief complaints by the patients and evolving symptoms/signs. Clearly if a patient presents early on with minor symptoms in the field, a paramedic may determine a patient did not need emergent evaluation. However, during the transportation and waiting in the ED for a bed, the patient’s condition might evolve into a more serious condition. By the time the patient is evaluated by a physician, the acuity status and/or chief complaint could drastically change through no fault of the paramedic or his/her training. Language barriers between the patients and paramedics may have also contributed to discrepancies between the acuity level evaluations. EPs have access to translation services that paramedics do not, which allows for additional information gathered on the patient’s chief complaint and medical history.

There is also the question of the difference in the definitions for acuity used by physicians and paramedics. While we attempted to use the same language for emergent, urgent and non-emergent/non-urgent by including these definitions on the surveys, either the physician or paramedic could have relied soley on experience when treating a patient presenting with a seemingly benign complaint that then resulted in a critical diagnosis made by the EP. Unfortunately, given that the paramedics’ job duties limit them to stabilizing and transporting patients to the ED for further evaluation, there is little opportunity for them to learn whether the patients ended up going home without any diagnostic testing or if their condition further deteriorated in the ED.

Lastly, although paramedics and physicians may have disagreed on their initial evaluations of patients, this may not have correlated with actual patient outcomes. No patient identifiers were included on either form completed by paramedics and physicians. This prevented tracking of a patient’s hospital course, admission versus discharge, and overall determination of the actual etiology and acuity of the patient’s chief complaint.

## CONCLUSION

This pilot study demonstrates that there is a significant difference in paramedics’ and physicians’ assessment of patients into emergent, urgent, or non-emergent/non-urgent categories. Targeted education on field triage, strict protocols, direct supervision with medical monitors and utilization of telemedicine may improve EMS providers’ triage diagnostic ability. Additionally, supervision by emergency physicians using new technologies, such as telemedicine, and a resolution to the isstue of lack of language translation services in the field may also improve paramedics’ triage of patients.

## Figures and Tables

**Table 1:** Comparison of acuity assessment by emergency physicians and paramedics.

	EP assessment emergent	EP assessment urgent	EP assessment non-emergent/non-urgent	Column total	P-value
Paramedics assessment emergent	224 (89.2%)	25 (10%)	2 (0.8%)	251	<0.0001
Paramedics assessment urgent	62 (34.8%)	98 (55.1%)	18 (10.1%)	178	
Paramedics assessment non-emergent/non-urgent	10 (13.5%)	25 (33.8%)	39 (52.7%)	74	
Row total	296	148	59	503	

*EP,* emergency physician.

Overall agreement between paramedics’ and EPs’ assessment on patients’ acuity level was 71.8% (224+98+39= 361 of 503, 71.8%).

Overall over-triage between paramedics’ and EPs’ assessment on patients’ acuity level was 8.9% (25+2+18= 45 of 503, 8.9%).

Overall under-triage between paramedics’ and EPs’ assessment on patients’ acuity level was 19.3% (62+10+25= 97 of 503, 19.3%).

** inter-rater Kappa=0.5174 between paramedics’ and EPs’ assessment on the same cohort of patients (n=503)

**Table 2:** Cases of over-triage* between paramedics’ and emergency physician’s assessment of patient’s acuity level.

EMS system	Frequency (N=45)	Percent
Neurological	10	22.2%
Musculoskeletal	8	17.8%
Cardiovascular	6	13.3%
Gastrointestinal	5	11.1%
Psychiatric	4	8.9%
Toxicological	4	8.9%
Endocrine	3	6.7%
Allergic/immunologic	2	4.4%
Respiratory	2	4.4%
HEENT	1	2.2%

* Over-triage was defined as higher acuity assessed by paramedics but lower acuity by emergency physician.

*EMS,* emergency medical services; *HEENT,* head eyes ears neck throat

**Table 3:** Cases of under-triage* between paramedics’ and EP’s assessment of patient’s acuity level.

EMS system	Frequency (N=97)	Percent
Musculoskeletal	25	25.8%
Gastrointestinal	20	20.6%
Neurological	14	14.4%
Cardiovascular	13	13.4%
Respiratory	7	7.2%
Endocrine	6	6.2%
Psychiatric	5	5.2%
Toxicological	4	4.1%
Allergic/immunologic	2	2.1%
Dermatological	1	1%

* Under-triage was defined as lower acuity assessed by paramedics but higher acuity by ED physician.

*EP,* emergency physician; *EMS,* emergency medical services

**Table 4:** Cases of agreement* between paramedic’s and emergency physician’s assessments of patient’s acuity level

EMS system	Frequency (N=361)	Percent
Neurological	73	20.2%
Musculoskeletal	68	18.8%
Cardiovascular	59	16.3%
Gastrointestinal	54	15%
Respiratory	30	8.3%
Toxicological	24	6.7%
Endocrine	20	5.5%
Psychiatric	19	5.3%
Dermatological	6	1.7%
Allergic/immunologic	5	1.4%
HEENT	3	0.8%

* Agreement was defined as same acuity assessed by paramedics and emergency physician.

*EMS,* emergency medical services; *HEENT,* head eyes ears neck throat
